# A unique *Helicobacter pylori* strain to study gastric cancer development

**DOI:** 10.1128/spectrum.02163-24

**Published:** 2024-12-06

**Authors:** Jeannette M. Whitmire, Ian H. Windham, Morris O. Makobongo, Mandy D. Westland, Sirena C. Tran, Jaume Piñol, Yvonne Hui, Rasha Raheem Alkarkoushi, Oscar Q. Pich, David J. McGee, M. Blanca Piazuelo, Angela Melton-Celsa, Traci L. Testerman, Timothy L. Cover, D. Scott Merrell

**Affiliations:** 1 Uniformed Services University of the Health Sciences, Bethesda, Maryland, USA; 2 University of South Carolina School of Medicine, Columbia, South Carolina, USA; 3 Vanderbilt University, Nashville, Tennessee, USA; 4 Institut de Biotecnologia i Biomedicina, Universitat Autònoma de Barcelona, Cerdanyola del Vallès, Spain; 5 Departament de Bioquímica i Biologia Molecular, Universitat Autònoma de Barcelona, Cerdanyola del Vallès, Spain; 6 Laboratori de Recerca en Microbiologia i Malalties Infeccioses, Hospital Universitari Parc Taulí, Institut d’Investigació i Innovació Parc Taulí (I3PT-CERCA), Universitat Autònoma de Barcelona, Sabadell, Spain; 7 Department of Microbiology and Immunology, LSU Health Sciences Center-Shreveport, Shreveport, Louisiana, USA; 8 Vanderbilt University Medical Center, Nashville, Tennessee, USA; 9 Veterans Affairs Tennessee Valley Healthcare System, Nashville, Tennessee, USA; 10 School of Animal and Comparative Biomedical Sciences, University of Arizona, Tucson, Arizona, USA; Ludwig-Maximilians-Universitat, München, Germany

**Keywords:** *H. pylori*, Mongolian gerbils, gastric adenocarcinoma, CagA, VacA

## Abstract

*Helicobacter pylori* colonizes a majority of the human population worldwide and can trigger development of a variety of gastric diseases. Since the bacterium is classified as a carcinogen, elucidation of the characteristics of *H. pylori* that influence gastric carcinogenesis is a high priority. To this end, the Mongolian gerbil infection model has proven to be an important tool to study gastric cancer progression. However, only a small number of *H. pylori* strains have been evaluated in the gerbil model. Thus, to identify additional strains able to colonize and induce disease in this model, several *H. pylori* strains were used to infect Mongolian gerbils, and stomachs were harvested at multiple timepoints to assess colonization and gastric pathology. The USU101 strain reproducibly colonized Mongolian gerbils and induced gastric inflammation in the majority of the animals 1 month after infection. Adenocarcinoma or dysplasia was observed in the majority of gerbils by 2 months post-infection. To define the contribution of key virulence factors to this process, isogenic strains lacking *cagA* or *vacA*, along with restorant strains containing a wild-type (WT) copy of the genes, were studied. The Δ*cagA* USU101 strain colonized gerbils at levels similar to WT, but did not induce comparable levels of inflammation or disease. In contrast, the Δ*vacA* USU101 strain did not colonize gerbils, and the stomach pathology resembled that of the mock-infected animals. The restorant USU101 strains expressed the CagA and VacA proteins *in vitro*, and *in vivo* experiments with Mongolian gerbils showed a restoration of colonization levels and inflammation scores comparable to those observed in WT USU101. Our studies indicate that the USU101 strain is a valuable tool to study *H. pylori*-induced disease.

**IMPORTANCE:**

Gastric cancer is the fifth leading cause of cancer-related death globally; the majority of gastric cancers are associated with *Helicobacter pylori* infection. Infection of Mongolian gerbils with *H. pylor*i has been shown to result in induction of gastric cancer, but few *H. pylori* strains have been studied in this model; this limits our ability to fully understand gastric cancer pathogenesis in humans because *H. pylori* strains are notoriously heterogenous. Our studies reveal that USU101 represents a unique *H. pylori* strain that can be added to our repertoire of strains to study gastric cancer development in the Mongolian gerbil model.

## INTRODUCTION

*Helicobacter pylori* chronically colonizes 50% of the world’s population, potentially causing a range of gastric diseases, including peptic ulcers and gastric cancer (1). Infection with *H. pylori* is a significant risk factor for the development of gastric cancer, which led to its classification as a Class I carcinogen (2, 3). Within the stomach, *H. pylori* interacts with host cells and produces various virulence factors that aid in the establishment of infection and disease progression. Understanding the contribution of *H. pylori*’s various virulence factors to disease etiology is vital to combating *H. pylori*-induced disease.

CagA and VacA are two extensively studied virulence factors within the *H. pylori* repertoire. Both proteins interact with host cells and significantly disrupt normal cell functions (4–8). VacA is secreted by the bacterium and forms membrane pores in host cells; intoxicated cells exhibit altered epithelial barrier function, disrupted mitochondria, and apoptosis (4–7). *H. pylori* delivers CagA into host cells through the Cag Type IV secretion system, resulting in alterations to numerous host cell signaling pathways (4, 5, 7–10). The extensive disruption of normal cell functions initiates the development of *H. pylori*-induced gastric pathologies. Indeed, multiple epidemiology studies have shown that infection with *cagA*-positive *H. pylori* strains is associated with more severe disease outcomes than infection with strains lacking *cagA (*9–13). Additionally, CagA is able to induce cancer when expressed in transgenic animals; thus, it is considered a bacterial oncoprotein (14). Taken together, the evidence from these studies suggests that VacA and CagA directly contribute to disease progression induced by *H. pylori*; however, more research is needed to dissect the specific roles of these virulence factors during disease development.

Animal infection models provide a significant resource to analyze the process of bacterial colonization and the development of disease *in vivo*. Infection models with *H. pylori* include mice, Mongolian gerbils, gnotobiotic piglets, and rhesus monkeys (15–21); animal models for various *H. pylori* strains have also recently been reviewed by Ansari and Yamaoka (22). In particular, the Mongolian gerbil model provides a stomach environment that closely mimics the conditions found in the human gastric environment, and experimental infection with *H. pylori* induces the development of gastric cancer (23–25). Indeed, Mongolian gerbils reproducibly develop adenocarcinoma lesions 8–12 weeks following infection with the 7.13 strain, which was originally isolated from a human as a strain designated B128, and then passaged in a gerbil to yield the 7.13 *H*. *pylori* strain (26, 27). Of note, B128 does not induce cancer in this model (26), and the molecular mechanisms by which *in vivo* passage of this strain resulted in the carcinogenic potential of the 7.13 strain remain unclear. While the 7.13 strain provides an excellent resource to study carcinogenesis for the *H. pylori* field, it is notable that there are no published animal studies using complemented or restorant studies derived from strain 7.13. The absence of such studies may be due to the loss of virulence exhibited by the 7.13 strain following *in vitro* passage (K.R. Jones and D.S. Merrell, unpublished results). Additionally, the 7.13 strain does not produce the VacA protein (28), since its coding sequence is truncated, thereby limiting the conclusions that can be raised on *in vivo* studies with this strain. Based on these challenges, the identification of another *H. pylori* strain that can reproducibly induce gastric cancer in a relatively short time frame within the Mongolian gerbil model would further the study of *H. pylori*-induced gastric cancer induction and progression. Efforts to identify additional *H. pylori* strains that cause gastric cancer in the gerbil would also be valuable because individual strains isolated from unrelated humans exhibit a high level of genetic diversity and can be considered genetically unique.

Herein, we characterize the USU101 strain and its ability to induce adenocarcinoma development in the Mongolian gerbil model. USU101 was originally isolated from a patient with gastric adenocarcinoma (16), and it has been utilized to investigate gastric cancer development in a rhesus macaque infection model (16). The presented data indicate that USU101 is a unique *H. pylori* strain that can be passaged *in vitro* without a loss of virulence; thus, this strain can be genetically manipulated and used to investigate gastric disease progression following infection with the bacterium.

## RESULTS

### Infection of gerbils with a panel of *H. pylori* strains including USU101 and J166

To preliminarily assess the ability of *H. pylori* strains to induce cancer in the Mongolian gerbil model of infection, gerbils were individually infected with a small panel of *H. pylori* strains, including J166, DSM719, DU3, G1, GU12, and USU101 (Table 1). At 3 months post-infection, strains J166 and USU101 colonized at levels similar to those previously observed in gerbils infected with 7.13 (29, 30) (data not shown), and the harvested stomachs from some of the gerbils infected with these strains displayed signs of adenocarcinoma (data not shown). While the other four strains included in the preliminary study also colonized gerbils at 3 months, bacterial levels were lower, and the gross pathological changes in the stomachs were not as drastic as those initiated after infection with the USU101 or J166 strains (data not shown). Thus, these two strains were chosen for a longer more thorough infection study.

**TABLE 1:** Strains used in this study

Strain	Merrell Lab strain designation	Description	Reference
USU101	DSM1367	WT *H. pylori*	(16, 31)
J166	DSM46	WT *H. pylori*	(32, 33)
DU3	DSM1267	Mouse-passaged J68 *H. pylori* strain	This study
G1	DSM1268	Mouse-passaged J178 *H. pylori* strain	This study
GU12	DSM1269	Mouse-passaged B134A *H. pylori* strain	This study
DSM719	DSM719	G27 *cagA*-AB^T^D	(34)
G27	DSM1	WT *H. pylori*	(35)
60190		WT *H. pylori*	ATCC 49503
26695	DSM18	WT *H. pylori*	(36)
DSM2076	DSM2076	USU101 Δ*cagA*::*kan,* Kan^R^	This study
DSM2079	DSM2079	USU101 *cagA:cat*, *cagA* restorant, Cm^R^	This study
DSM2086	DSM2086	USU101 Δ*vacA*::*kan,* Kan^R^	This study
DSM2088	DSM2088	USU101 *vacA:cat*, *vacA* restorant, Cm^R^	This study

**Fig 1:**
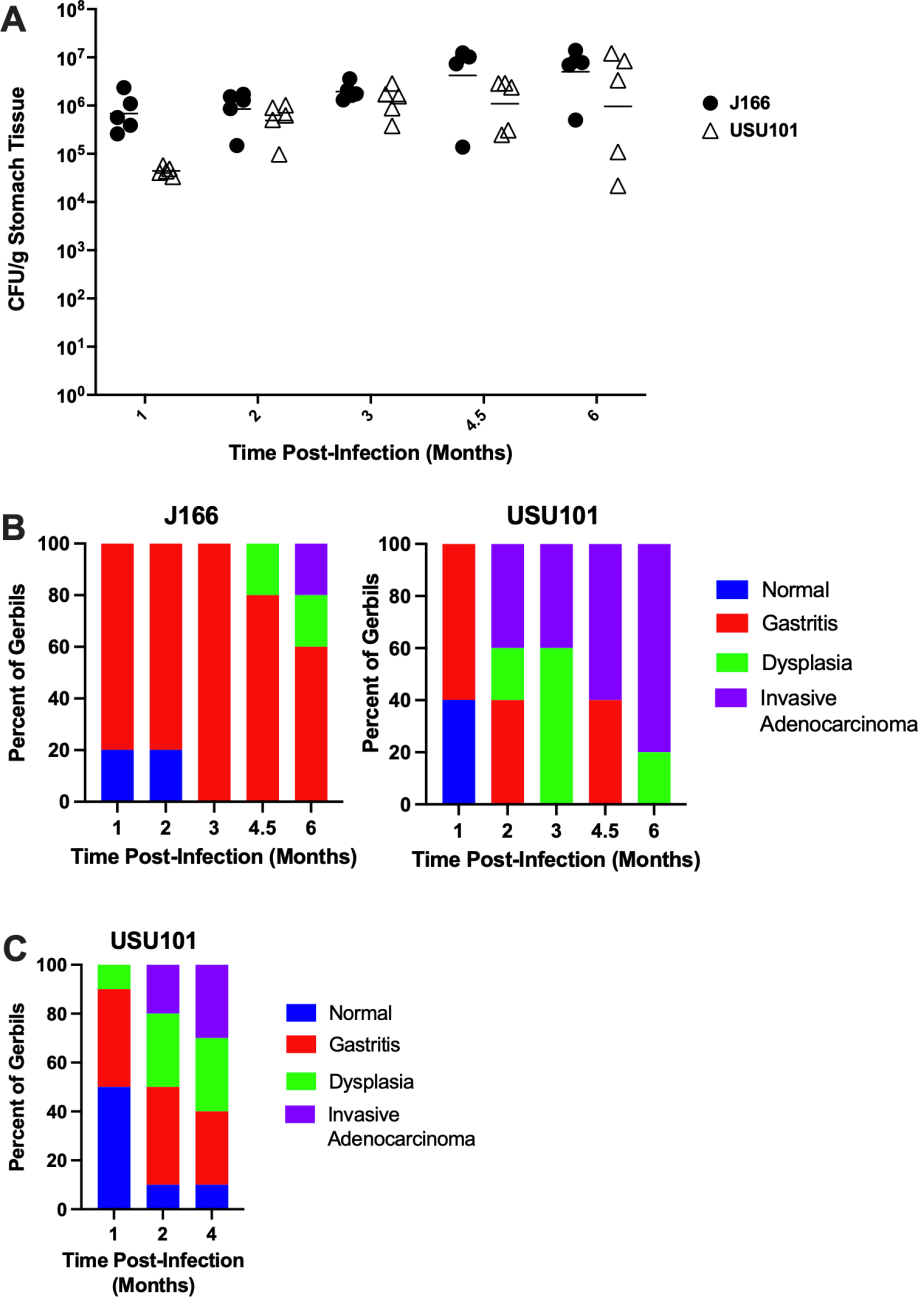
Gerbil infections with USU101 and J166. (**A**) Colonization levels (CFU/g stomach tissue) of J166 (black circles) and USU101 (open triangles) at five timepoints post-infection. (**B**) Percent of gerbils (*n* = 5) displaying the indicated gastric histologic diagnoses for each strain at five timepoints post-infection. (**C**) Percent of gerbils (*n* = 10) displaying the indicated gastric histologic diagnoses for USU101 at three timepoints post-infection in a study conducted at a different institution (University of South Carolina School of Medicine).

In the subsequent infection study, Mongolian gerbils were orogastrically infected with either J166 or USU101, and the stomachs were harvested at 1, 2, 3, 4.5, or 6 months post-infection. The harvested stomachs were assessed for colonization levels and also analyzed for pathological changes. Both strains successfully colonized all of the animals for the duration of the study. Though USU101 appeared to colonize at a reduced level 1 month post-infection, both strains chronically colonized the gerbil stomachs in the subsequent months at levels similar to those previously observed during infections with 7.13 (29, 30) (Fig. 1A). This finding confirmed that these strains can establish a chronic infection in the Mongolian gerbil model. Moreover, the pathology analysis of the gerbil stomachs confirmed gastric disease (gastritis, dysplasia, or invasive adenocarcinoma) development. In gerbils infected with J166, 80% of the animals developed gastritis by 1 month post-infection, and a similar proportion had gastritis at 2 months post-infection (Fig. 1B). By 3 months post-infection, all animals displayed gastric disease. At 4.5 months post-infection, 20% of the animals exhibited dysplasia, and 80% had gastritis. By 6 months post-infection, 20% of the gerbils exhibited dysplasia, 20% displayed adenocarcinoma, and 60% had gastritis (Fig. 1B). Thus, J166 appeared to have the ability to induce gastric cancer, but this only occurred in one animal and not until 6 months post-infection. Strain USU101 also caused gastritis in gerbils at 1 month post-infection. By 2 months post-infection, USU101 caused gastric disease in all infected animals with 20% exhibiting dysplasia, 40% displaying adenocarcinoma, and 40% having gastritis (Fig. 1B). Thus, by 2 months post-infection, USU101 induced the development of gastric cancer in this model. By 6 months post-infection, 80% of the gerbils had adenocarcinoma, and the remaining 20% had dysplasia (Fig. 1B). A similar infection study using USU101 at a separate institution (University of South Carolina School of Medicine) further supported our data, with 50% of gerbils displaying gastric disease (gastritis or dysplasia) 1 month post-infection, 55% of animals exhibiting dysplasia or adenocarcinoma 2 months post-infection, and 60% of animals with dysplasia or adenocarcinoma 4 months post-infection (Fig. 1C).

### Sequencing of USU101 genome

To further characterize the USU101 strain, the genome was sequenced using the Illumina platform. The sequencing resulted in a 1.7 Mb genome, with a total of 1,671 genes, including 1,512 genes coding for proteins, 113 pseudogenes, and 46 RNA coding genes. As previously characterized, USU101 is *cagA*-positive with an ABC EPIYA motif and an s1/i1/m1 *vacA* allele (16). Genome sequencing confirmed these prior findings. The annotated genomic sequence is available through the National Center for Biotechnology Information (NCBI) Bioproject PRJNA494865.

### *In vitro* analysis of USU101 mutant and restorant strains

To identify the contribution of specific virulence factors to the ability of USU101 to colonize and cause disease, mutant strains that lacked *cagA* and *vacA* were constructed. To ensure that any observed phenotypes were a result of the missing virulence factor genes, restorant strains were also constructed to replace the deleted genes at their respective native locus. Transformation efficiency varies greatly among different *H. pylori* strains and is often limited by restriction-modification systems (37–41). As such, USU101 proved to be a strain that was challenging to transform via natural transformation. While construction of complementation strains would have been ideal from a genetic perspective, multiple attempts to utilize the IR0203-IR0204 genomic region (42) or the *rdxA* locus (43) did not readily provide the desired strains; we did not pursue plasmid-based complementation approaches due to concerns about *in vivo* plasmid loss in the absence of selective antibiotic pressure. However, we were able to successfully create restorant strains where the individual mutations were replaced by a USU101 WT copy of the mutated gene at the native locus. Thus, while USU101 was not as genetically tractable as some standard *H. pylori* strains, we were able to construct the following strains in the USU101 background: Δ*cagA*, Δ*vacA*, *cagA* restorant, and *vacA* restorant.

**Fig 2:**
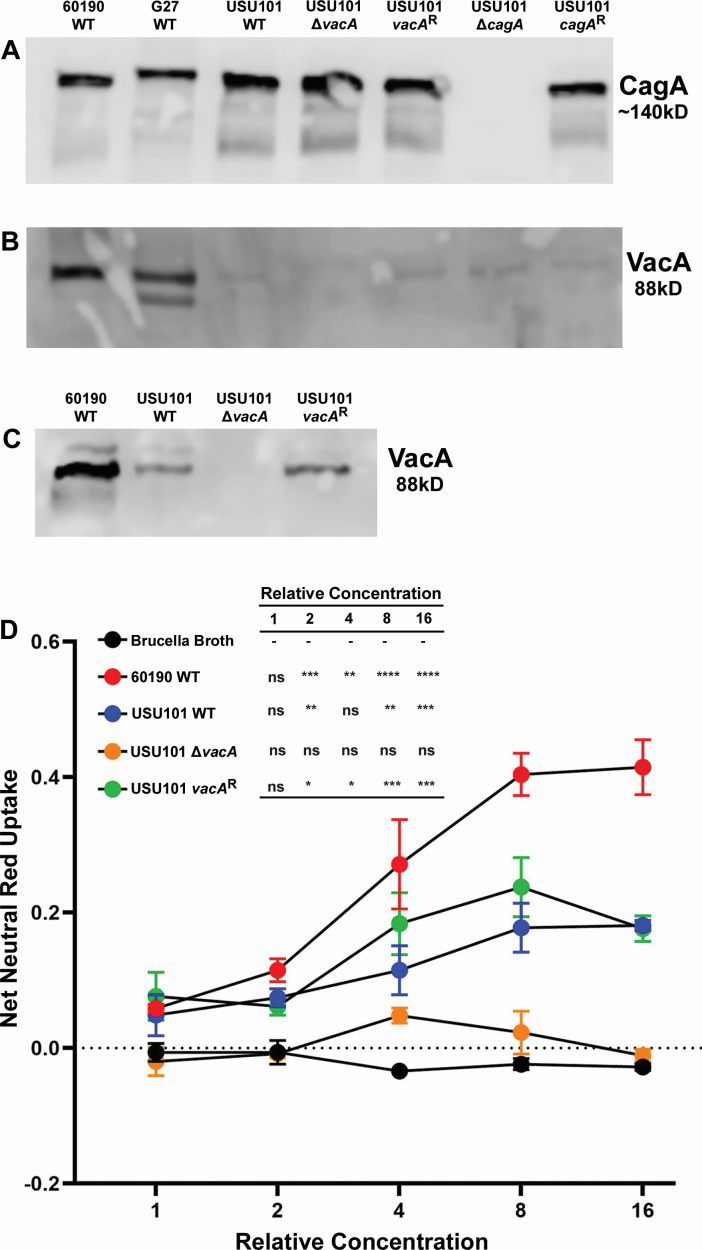
Western blot analysis. (**A**) CagA Western blot of the indicated strains. WT 60190 and WT G27 strains are shown for comparison. (**B**) VacA Western blot of the indicated strains. WT 60190 and WT G27 strains are shown for comparison. (**C**) VacA Western blot of broth culture supernatants from the indicated strains. The WT 60190 strain was included for comparison. (*cagA*^R^ = *cagA* restorant strain; *vacA*^R^ = *vacA* restorant strain). (**D**) Cell vacuolation induced by broth culture supernatants from the indicated strains as indicated by neutral red uptake. The inset shows statistical significance as determined by an ordinary one-way analysis of variance with a Dunnett’s multiple comparison test. The Brucella broth control at each of the relative concentrations was used as the comparison group to each of the other indicated strains and the symbols denote the following: -- the comparator group, ns no significance, * *P* < 0.05, ** *P* < 0.005, *** *P* < 0.0005, and **** *P* < 0.0001.

Western blot analysis of the constructed strains was utilized to ensure that the strains produced or failed to produce the appropriate proteins (CagA or VacA). Western blots using lysates from each strain revealed that all the strains except the Δ*cagA* strain produced the CagA protein (Fig. 2A). Similarly, although the levels were greatly reduced compared to other lab strains, including G27 and 60190 (Fig. 2B), the VacA protein was detected in all strains except the Δ*vacA* strain. Further analysis of the *vacA*-manipulated strains via examination of supernatants from *H. pylori* broth cultures confirmed the production and secretion of VacA by the WT and restorant *vacA* strains; the Δ*vacA* strain did not produce or secrete VacA (Fig. 2C). Additionally, assays to assess protein functionality confirmed the vacuolation ability of VacA in WT USU101, although the level of vacuolation was lower than that produced by a control strain that produces high levels of VacA (WT 60190) (Fig. 2D). As expected, the Δ*vacA* strain did not produce vacuolation above background levels, while the restorant *vacA* strain displayed a level of vacuolation similar to that caused by WT USU101 (Fig. 2D). These data verified that the mutant and restorant strains exhibited the expected protein phenotypes.

**Fig 3:**
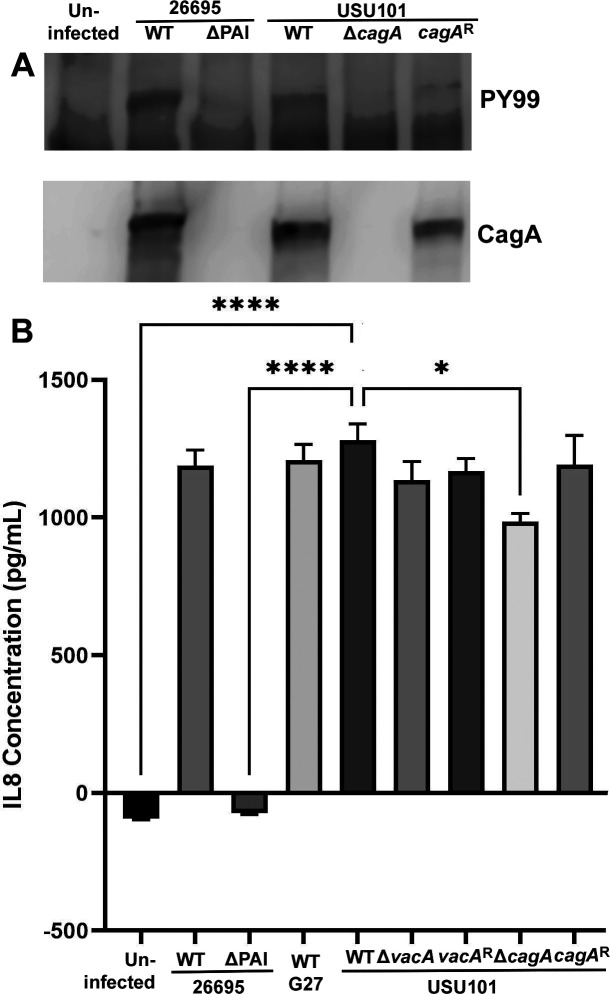
CagA translocation and IL-8 induction. (**A**) Western blots of AGS cells infected with the indicated strains and probed with PY99 to detect phosphorylated CagA and anti-CagA to detect total CagA. The 26695 WT strain and 26695 ∆*cag* PAI mutant are included for comparison. (**B**) Induction of IL-8 production by AGS cells following infection with the indicated strains. The *y*-axis shows IL-8 concentrations in supernatants from AGS cells. WT 26695, 26695 ∆*cag* PAI mutant, and WT G27 were included for comparison. Statistical significance was analyzed by comparing each group to WT USU101 using a one-way analysis of variance with a Dunnett’s multiple test correction; significant differences are noted on the graph. (*cagA*^R^ = *cagA* restorant strain; *vacA*^R^ = *vacA* restorant strain) (*P < 0.05; ****P < 0.0001).

Next, CagA translocation was examined by analyzing the WT and *cagA*-manipulated strains in an AGS gastric epithelial cell culture assay. Phosphorylated CagA was identified in lysates from host cells infected with the WT and *cagA* restorant USU101 strains (Fig. 3A), indicating the ability of the strains to produce and translocate CagA. As expected, the Δ*cagA* strain did not produce detectable CagA, and no phosphorylated band at the size of CagA appeared on the blot (Fig. 3A). The amount of CagA produced and translocated by USU101 was at a much lower level as compared to that produced by a control strain commonly used for CagA translocation assays (strain 26695).

Interleukin (IL)-8 induction was also assessed to further validate the phenotypes of the mutant and restorant strains. The 26695 wild-type (WT) strain and a 26695 ∆*cag* PAI mutant strain (∆PAI), which bears a deletion of the *cag* pathogenicity island that encodes the Type IV secretion system needed to deliver CagA to host cells, were included for comparison. As expected, all strains, except ∆PAI, were able to induce IL-8 production in the infected cells (Fig. 3B). The Δ*cagA* strain induced IL-8 levels at a statistically significant lower level compared to WT USU101. Collectively, the *in vitro* data indicated that the mutant and restorant strains behaved as expected.

### *In vivo* characterization of mutant and restorant USU101 strains

**Fig 4:**
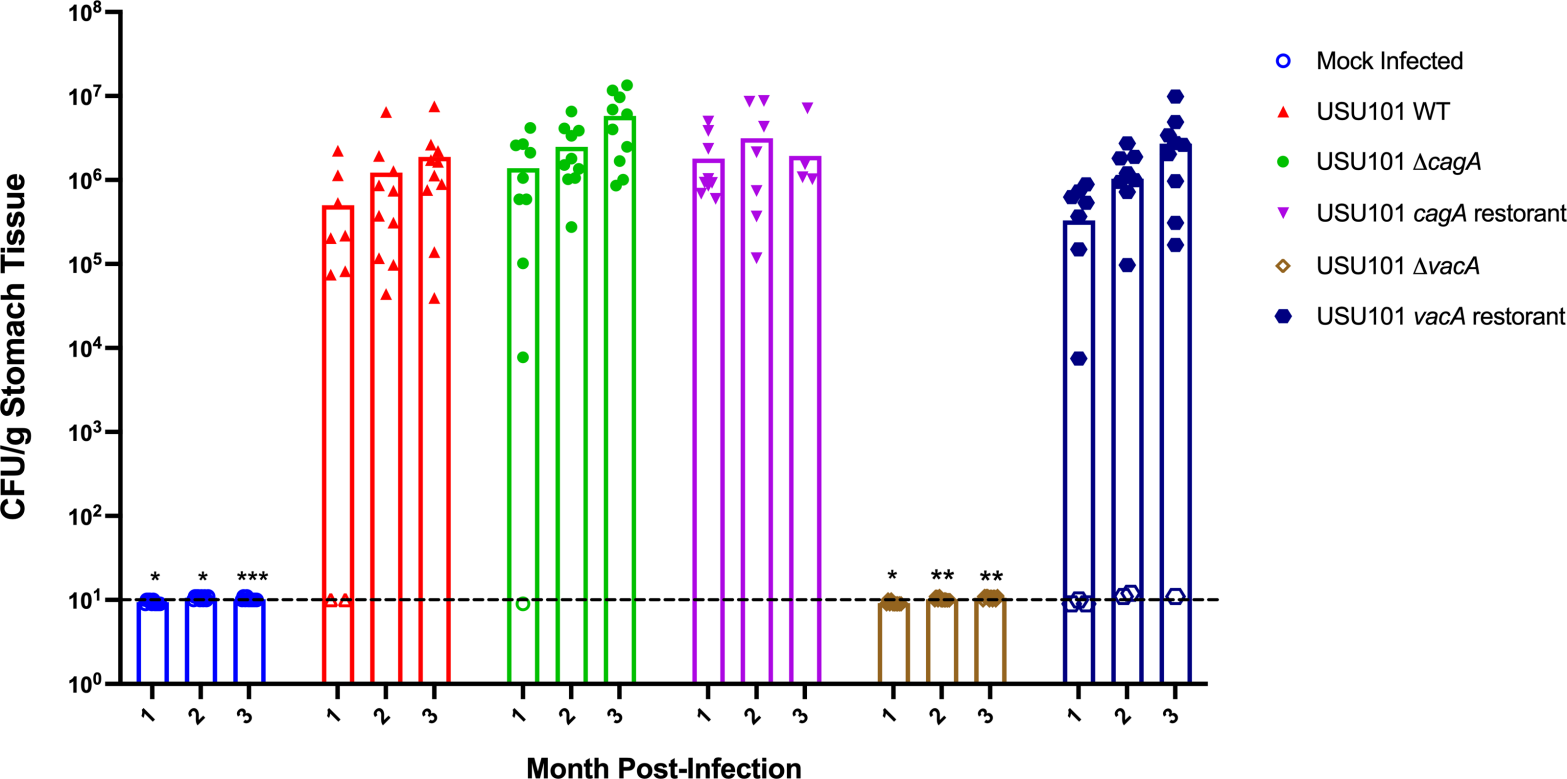
Colonization levels of USU101 and isogenic strains. Colonization levels (CFU/g stomach tissue) for the indicated strains are plotted at the indicated timepoints post-infection. Each point indicates the colonization level of an individual gerbil, and the bars indicate the mean for the animals in the group at each timepoint. The dashed line demarcates the limit of detection (LOD) of 10–15 CFU, and non-colonized gerbils are plotted at the LOD. Statistical significance at each timepoint relative to the USU101 WT colonization level at the same timepoint was assessed using the Kruskal–Wallis test with Dunn’s correction for multiple comparisons. Significant differences are indicated as follows: * *P* < 0.05, ** *P* < 0.01, *** P ≤ 0.001.

Following successful *in vitro* characterization, the mutant and restorant USU101 strains were next used to infect Mongolian gerbils; positive control animals were infected with the WT USU101 strain, while the mock-infected control group was dosed with phosphate buffered saline. Two biologically independent experiments were conducted (*n* = 4–5 gerbils/group/experiment). Stomachs from all six infection groups were harvested at 1, 2, or 3 months post-infection for colonization assessment and pathology determination. At 1, 2, or 3 months post-infection, the Δ*cagA* and *cagA* restorant strains colonized the gerbils at levels similar to the USU101 WT strain, which were comparable to the levels observed in previous infections with USU101 (Fig. 1 and 4). In contrast, the Δ*vacA* strain did not colonize at any of the analyzed timepoints; however, the *vacA* restorant strain colonized the gerbils at levels similar to the USU101 WT strain (Fig. 4). These data indicate that CagA is not required for colonization of USU101; however, VacA was required for colonization of the gerbils.

**Fig 5:**
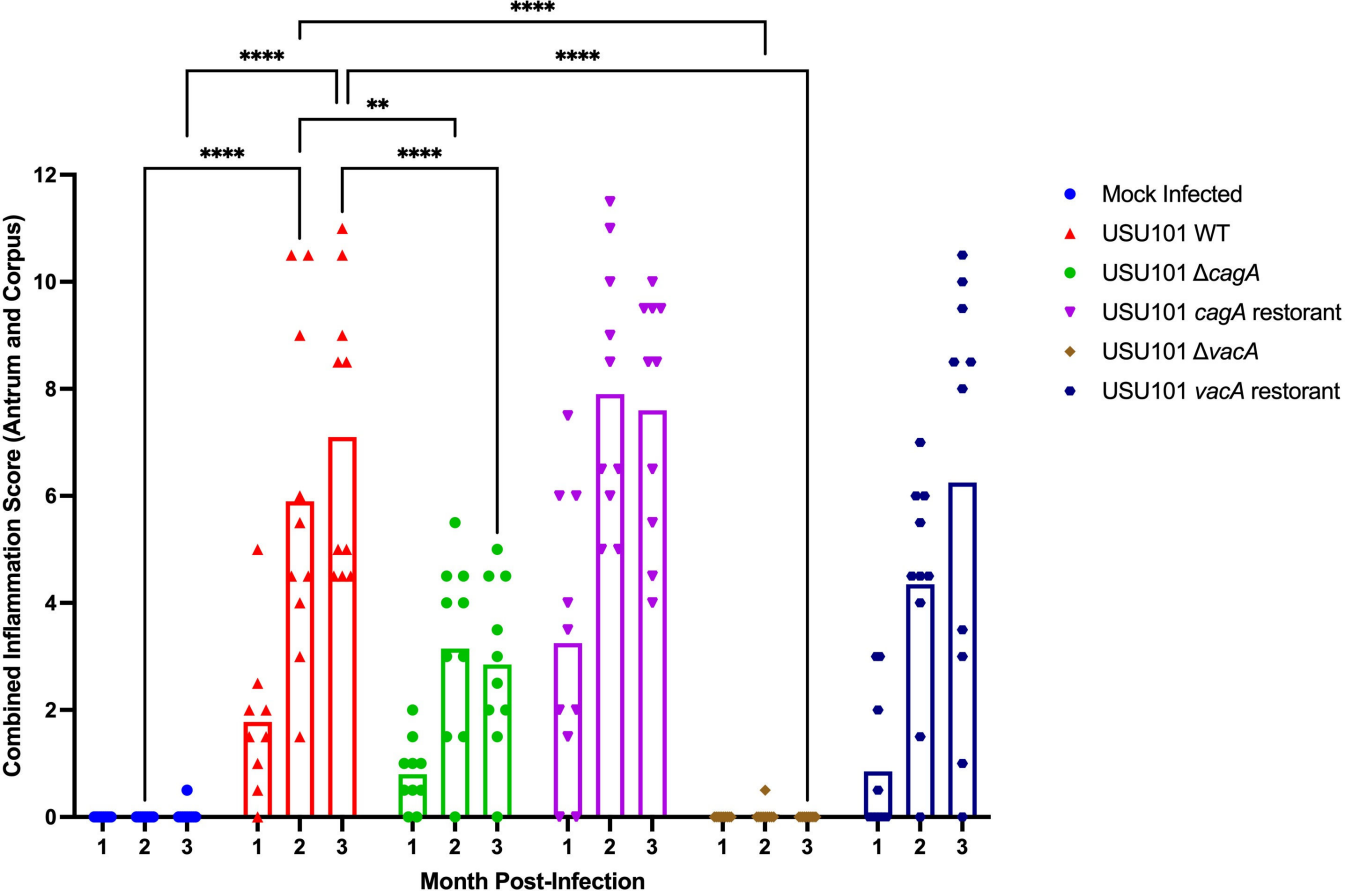
Inflammation scores. Inflammation scores were generated by combining the scores obtained from the antrum and corpus and are plotted for the indicated strains at each of the noted timepoints post-infection. Each point indicates the combined inflammation score for an individual gerbil, and the bars indicate the mean for the animals in the group at each timepoint. A two-way analysis of variance with multiple comparisons was performed to identify statistically significant differences between groups. (**P < 0.01; ****P < 0.0001).

Next, the stomach tissue from the infected gerbils was graded for inflammation in both the antrum and corpus. At 1 month post-infection, the gerbils infected with the USU101 WT, Δ*cagA*, *cagA* restorant, and *vacA* restorant strains exhibited inflammation, while the mock-infected and Δ*vacA*-infected animals did not display inflammation (Fig. 5). Though the gerbils exhibited varying levels of inflammation or lacked inflammation, none of the groups had gastric inflammation significantly different compared to the inflammation induced by the USU101 WT strain at this timepoint. At 2 months post-infection, the gerbils infected with the USU101 WT, Δ*cagA*, *cagA* restorant, and *vacA* restorant strains exhibited gastric inflammation; however, the combined inflammation score for the Δ*cagA*-infected gerbils was significantly lower than that of animals infected with the WT strain (Fig. 5).

Similarly, the absent and reduced inflammation observed in mock-infected and Δ*vacA*-infected animals, respectively, was statistically significant compared to that observed in the gerbils infected with the WT strain. At the 3 month timepoint, the *cagA* restorant and *vacA* restorant strains induced similar levels of inflammation compared to the WT strain, while the Δ*cagA* strain caused significantly reduced levels of inflammation (Fig. 5). The minimal level of inflammation in the mock-infected group and the absence of inflammation in the Δ*vacA*-infected animals were statistically significant when compared to the WT group.

**Fig 6:**
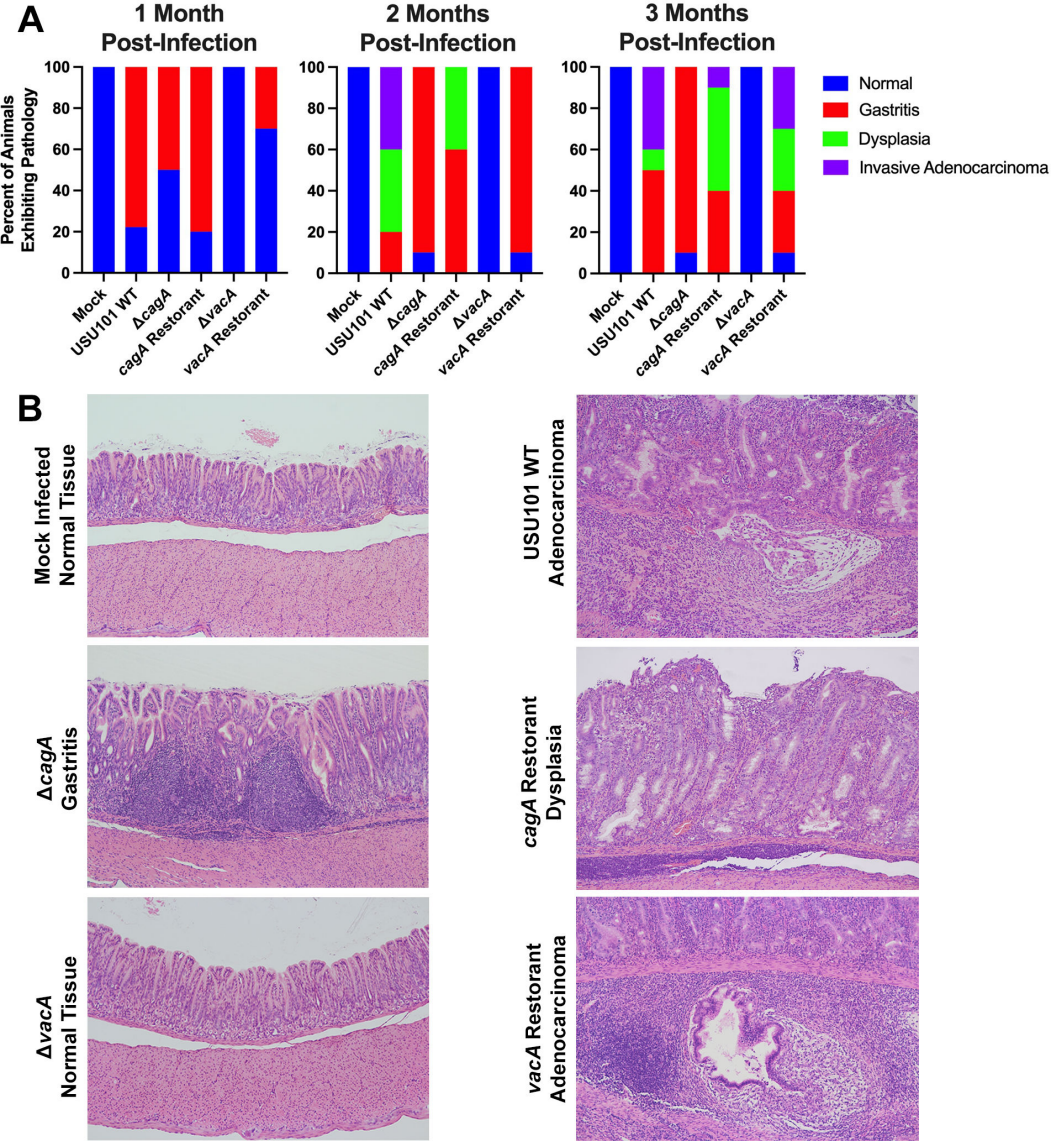
Gastric pathology determination. (**A**) Percent of gerbils (*n* = 10) displaying the indicated gastric histologic diagnoses for each strain at three timepoints post-infection. (**B**) Representative images of stomach tissue stained with hematoxylin and eosin for each strain at 3 months post-infection. All images were taken at 100× magnification.

The infected stomach tissue was also analyzed to identify the presence of gastric disease. The gastric histology was categorized into four diagnostic categories: normal, gastritis, dysplasia, and invasive adenocarcinoma. Similar to the data generated in our initial experiments (data not shown and Fig. 1B and C), gastric disease (defined as any diagnosis other than normal) was observed in all but one of the gerbils infected with USU101 WT at 1 month post-infection. In contrast, the stomach tissue of mock-infected gerbils exhibited no evidence of gastric disease at any timepoint (Fig. 6A). Gastric disease was noted in all gerbils infected with USU101 WT at both the 2 month and 3 month timepoints, with dysplasia or adenocarcinoma occurring in 80% of gerbils 2 months post-infection and in 50% of animals at the 3 month timepoint (Fig. 6A). Adenocarcinoma is characterized by the presence of multiple irregular, dysplastic glands showing submucosal invasion, and a representative example is displayed in Fig. 6B, while the normal gastric appearance of the stomach tissue is evidenced in Fig. 6B. Gerbils infected with the *cagA* mutant did not exhibit dysplasia or adenocarcinoma at any of the timepoints, although gastritis was noted in 50% of the animals 1 month post-infection and 90% of the gerbils at the 2 and 3 month timepoints (Fig. 6A). Gastritis is characterized by increased immune infiltrate in the mucosa, and a representative example of a gerbil stomach infected with the *cagA* mutant strain showing the formation of lymphoid follicles is included in Fig. 6B. In contrast to the *cagA* mutant, the animals infected with the *cagA* restorant strain showed evidence of dysplasia or adenocarcinoma in proportions that were similar to the WT strain: 40% with dysplasia at 2 months post-infection and 60% of gerbils with dysplasia or adenocarcinoma at the 3 month timepoint (Fig. 6A). Dysplasia is characterized by the presence of irregular and angulated glands, sometimes dilated, and spreading horizontally with respect to the muscularis mucosa; a representative example from the gerbils infected with the *cagA* restorant is exhibited in Fig. 6B. The *vacA* mutant failed to colonize any gerbils throughout the course of the study; thus, the stomach tissue was comparable to that of the mock-infected animals, and no evidence of gastric disease was noted at any timepoint (Fig. 6A and B). In contrast, gastritis was noted in 30% of gerbils infected with the *vacA* restorant at the 1 month timepoint and in 90% of animals 2 months post-infection. By the 3 month timepoint, all but one animal infected with the *vacA* restorant exhibited gastric disease, with 60% of the gerbils diagnosed with dysplasia or adenocarcinoma, which is at a level similar to the WT strain (Fig. 6A). A representative example of a gerbil stomach infected with the *vacA* restorant, which exhibited invasive adenocarcinoma, is included in Fig. 6B.

## DISCUSSION

While investigating the colonization and pathogenicity of *H. pylori* strains in the context of the Mongolian gerbil model, USU101 was identified as a novel strain that causes gastric disease progression, including adenocarcinoma. Indeed, the WT USU101 strain consistently colonized Mongolian gerbils at levels similar to the well-studied 7.13 strain (29, 30). Additionally, the majority of infected gerbils exhibited gastritis by 1 month post-infection, with dysplasia or adenocarcinoma appearing in the majority of the animals 2 months after infection. At 3 and 6 months after infection, all of the gerbils showed signs of dysplasia or adenocarcinoma. The rapid development of disease in the Mongolian gerbils makes USU101 an extremely useful strain for the study of gastric disease progression without the need for extended lengths of colonization.

Sequencing of the USU101 strain confirmed the previously reported EPIYA-ABC CagA type and s1/i1/m1 VacA type (16). To further characterize the USU101 strain, mutant strains were constructed to delete the *cagA* or *vacA* genes along with restorant strains to reinsert the genes into their native locus. The USU101 strain proved to be more difficult to genetically manipulate than traditional lab-passaged strains; however, genetic manipulation was possible, and the mutant and restorant strains were constructed. This resistance to genetic manipulation may prove to be beneficial for its use in the Mongolian gerbil model as the strain should be less likely to incorporate foreign DNA or lose virulence with multiple passages; this strain has been passaged *in vitro* in the Merrell laboratory for many years and is clearly still virulent in the gerbil model.

As observed in other *H. pylori* strains and *in vivo* experiments, CagA was not required for colonization of Mongolian gerbils with the USU101 strain (27, 44, 45). However, the lack of CagA led to reduced inflammation and gastric pathologies as expected (27). Restoring the *cagA* gene to its native locus resulted in colonization, inflammation, and gastric pathology similar to the WT USU101 strain. These results further support the utility of USU101 as a strain to study gastric carcinogenesis; the strain can be genetically manipulated to create mutant strains and their respective restorant strains to establish a gene’s role in an observed phenotype.

Previous studies have not identified a requirement for VacA to establish colonization in the Mongolian gerbil model (19, 21, 27, 46). In fact, strain 7.13 does not produce a functional VacA protein (28) due to a frame-shift mutation in the coding sequence, yet the strain colonizes Mongolian gerbils. Some studies using a mouse model have observed reduced colonization levels of *vacA* mutants compared to their WT parent strain (47–49), while others have described similar colonization levels (50, 51); one study also noted that a *vacA* mutant exhibited competitive deficiencies and increased ID_50_ levels compared to the WT strain (51). In contrast, the USU101 strain lacking *vacA* failed to colonize Mongolian gerbils, while the VacA restorant strain was able to colonize and induce disease in the gerbils at levels similar to the WT USU101 strain. Of note, the prior colonization studies of defined *vacA* mutant strains relied on the utilization of strains containing insertions of an antibiotic marker within the coding sequence of *vacA* (19, 21, 27, 46). In contrast, we generated a strain containing a substantial deletion of *vacA*; only 405 nucleotides from the 5′ end of the coding sequence remain of the 3,888 total nucleotides of the *vacA* coding sequence in our Δ*vacA* strain. Thus, it is intriguing to speculate that a role for VacA, or the genetic region containing *vacA*, in colonization and disease may have been previously missed based on the utilized mutagenesis strategy. To this end, we note that transcription start sites for two small RNA’s have been annotated on the minus strand of the coding sequence for *vacA*: HPnc4480 and HPnc4490 (52, 53). The sequences for both of these sRNA’s would have remained intact in several of the prior studies (19, 21, 27), while these sequences were removed from the USU101 *vacA* mutant in this study. Perhaps these sRNA’s play an as-yet unidentified role during the course of colonization in the Mongolian gerbil. The requirement of VacA for colonization of USU101 in the Mongolian gerbil infection model presents an interesting avenue for future investigation.

Overall, the presented data indicate that USU101 is a useful strain for the study of *H. pylori*-induced gastric cancer development. The strain reproducibly induces gastric disease and adenocarcinoma in the Mongolian gerbil infection model within a relatively short time frame. Additionally, the strain can be genetically manipulated to assess the role of various genes during the course of *H. pylori* infection and disease development. Thus, the USU101 strain will provide an additional tool to further the study of *H. pylori*-induced gastric disease.

## MATERIALS AND METHODS

### Bacterial strains and growth

Table 1 includes a list of all strains used in this study. G1 is a mouse-passaged derivative of strain J178, which was originally isolated from a patient presenting with gastritis. DU3 is a mouse-passaged derivative of strain J68 that was originally isolated from a patient presenting with a duodenal ulcer. GU12 is a mouse-passaged derivative of strain B134A that was originally isolated from a patient presenting with a gastric ulcer. The original J178, J68, and B134A isolates were obtained from Dr. Richard Peek at Vanderbilt University and were minimally passaged (54–58).

*H. pylori* strains were maintained as frozen stocks at −80°C in brain heart infusion broth (BD, Franklin Lakes, NJ, USA) supplemented with 10% fetal bovine serum (Gibco, Waltham, MA) and 20% glycerol (Thermo Scientific, Waltham, MA). Bacterial strains were expanded on horse blood agar (HBA) plates comprised of 4% Columbia agar base (Neogen Corporation, Lansing, MI, USA), 5% defibrinated horse blood (HemoStat Laboratories, Dixon, CA, USA), 0.2% β-cyclodextrin (Millipore Sigma, Burlington, MA, USA), 10 µg/mL vancomycin (Amresco, Radnor, PA, USA), 5 µg/mL cefsulodin (Millipore Sigma), 2.5 U/mL polymyxin B (Millipore Sigma), 5 µg/mL trimethoprim (Millipore Sigma), and 8 µg/mL amphotericin B (Amresco). Liquid cultures of *H. pylori* were grown in Brucella broth (Neogen Corporation) supplemented with 10% fetal bovine serum and 10 µg/mL vancomycin at 37°C with shaking at 100 rpm. Plates were supplemented with 6 µg/mL chloramphenicol (Cm) (Millipore Sigma) or 25 µg/mL kanamycin (Kan) (Gibco) as noted in Table 1. Liquid and plate cultures were both incubated under microaerobic conditions (5% O_2_, 10% CO_2_, and 85% N_2_) generated with an Anoxomat gas evacuation and replacement system (Advanced Instruments, Norwood, MA, USA) in gas evacuation jars.

### Genome sequencing of USU101 strain

DNA from the *H. pylori* USU101 strain was purified using the Easy-DNA gDNA Purification Kit (Life Technologies, Waltham, MA, USA). Libraries were obtained with the Nextera Mate Pair Library Preparation Kit using the gel-plus protocol and selecting for fragments of approximately 8 kb. Samples were then submitted to an Illumina HiSeq system, which rendered about 3.5 million paired reads, each read containing sequences 125 nt long, with a negligible frequency of ambiguous bases. Data were processed with the NxTrim v0.4.3 tool (59) to remove the junction adapters and to categorize reads according to the orientation implied by the adapter location. Then, trimmed sequences were submitted to the Velvet assembler v1.1 (60) using a kmer size of 95 and the scaffolding option. The assembler returned a single scaffold containing 24 gaps. This scaffold was further refined by the Pilon tool v1.24 (61), obtaining a final assembly of 1,695,835 bp containing only three minor gaps. The final assembly was deposited to the NCBI (Bioproject PRJNA494865) and annotated by the standard NCBI Prokaryotic Genome Annotation Pipeline.

### Natural transformation of USU101

The USU101 strain was expanded on HBA plates from a frozen glycerol stock and allowed to grow for 3 days as described above. The expanded cells were retrieved using a sterile cotton swab and resuspended in 300 µL of Brucella broth. The resuspended cells were spread on a fresh HBA plate and allowed to grow for 1 day. Once light growth was observed, the cells were retrieved using a sterile cotton swab and resuspended in 1 mL of Brucella broth; successful transformation was only achieved when cells were captured in this early phase of “light“ growth on the plates. The cells were pelleted by centrifugation at 4.5K for 4 minutes at +4°C, and the pellet was resuspended in 50 µL of Brucella broth. The resuspended cells were gently pipetted on a fresh HBA plate and allowed to dry. The plate was placed in a 37°C incubator under microaerobic conditions for 5–6 hours. A 15 µL suspension containing the DNA for transformation was added to the spotted cells; 1.5–2 μg of DNA as described below was used for the transformations. Once the DNA suspension dried, the plate was returned to the 37°C incubator under microaerobic conditions for 18 hours. The resulting spot of cells was retrieved with a sterile cotton swab and resuspended in 1 mL of Brucella broth. The cells were pelleted by centrifugation at 4.5K for 4 minutes at +4°C, and the pellet was resuspended in 100 µL of Brucella broth. Individual aliquots of 10 µL and 90 µL were spread on separate antibiotic selection HBA plates, and the plates were placed in the 37°C incubator under microaerobic conditions for 5–8 days until individual colonies appeared. Individual colonies were retrieved using sterile 20 µL pipet tips and spread in a ¼ inch streak on an antibiotic selection HBA plate and allowed to grow for 2–3 days. The small streaks were retrieved with a sterile cotton swab wetted with sterile Brucella broth and expanded onto a portion of an antibiotic selection HBA plate and allowed to grow for 1–2 days. The lawned cells were then used for genomic DNA isolation and frozen glycerol stock preparation.

### Strain construction

All isogenic mutant and restorant strains were created using the USU101 strain background (16, 31). Primer sequences used in this study are listed in Table 2. To create the Δ*cagA* mutant strain, sequences approximately 1 kb upstream and downstream of the *cagA* coding sequence were amplified from USU101 genomic DNA along with the *aphA-3* gene from pUC18K-2 (62). The 1 kb sequence upstream of *cagA* was amplified with USU101delcagA_upF and USU101delcagA_upR. The 1 kb sequence downstream of *cagA* was amplified with USU101delcagA_downF and USU101delcagA_downR. The kanamycin-resistant cassette encoded by the *aphA-*3 gene was amplified from pUC18K-2 with USU101cagAdel_KanF and USU101cagAdel_KanR. The three amplicons were joined using splicing by overlap extension (SOE) such that the upstream and downstream fragments flanked the kanamycin-resistant cassette. The resulting amplicon was used to transform USU101 via natural transformation, and transformants were selected on HBA plates supplemented with 25 µg/mL kanamycin. The deletion of *cagA* was confirmed using primers USU101cagA_FarupF and USU101cagA_FardownR, which reside outside of the cloned region. The resulting strain was further confirmed by sequencing with primers USU101cagA_FarupF, KanR_RC, and USU101cagA_FardownR, and the strain was named DSM2076.

**TABLE 2:** Primers used in this study

Primer name	Sequence (5′ to 3′)
USU101cagAdel_upF	GCTATGCCTTTGACCCCTAGAGC
USU101cagAdel_upR	GTTAGTCACCCGGGTACCGAGCTCCATTGTTTCTCCTTACTAACCTAGTTTC
USU101cagAdel_downF	CTAGAGTCGACCTGCAGGCATGCAAGTAAAGGATTAAGGAATACCAAAAACGC
USU101cagAdel_downR	CTTGCATGCGTTATTATTTCACTCC
USU101cagAdel_KanF	GAAACTAGGTTAGTAAGGAGAAACAATGGAGCTCGGTACCCGGGTGACTAAC
USU101cagAdel_KanR	GCGTTTTTGGTATTCCTTAATCCTTTACTTGCATGCCTGCAGGTCGACTCTAG
USU101cagA_FarupF	GCGCGTAAGCAAAAACAGTCGCC
USU101cagA_FardownR	GCTATTTGAATGTTTCGCATTTGGCC
USU101_cagALoc_Comp_upR	CTCTCTGTCGAGAGTAGTGCGGCGTTTTTGGTATTCCTTAATCCTTTAAG
USU101_cagALoc_Comp_downF	CCACTATATCATAAATCTATCTAAAGGATTAAGGAATACCAAAAACGC
USU101_cagALoc_Comp_catF	CTTAAAGGATTAAGGAATACCAAAAACGCCGCACTACTCTCGACAGAGAG
USU101_cagALoc_Comp_catR	GCGTTTTTGGTATTCCTTAATCCTTTAGATAGATTTATGATATAGTGG
USU101_cagseq_nt960_F	GGGCAATGGTGGTTTTGGAGCCAAG
USU101_cagseq_nt1400_R	CTCAAATCCCCATTACCAAACTCAG
USU101CagA1020R	CCCAACTTGTGAAAATTTGGTAACGC
USU101CagA1960F	CGCCCTAGGGAATGATCG
USU101cagAdel_upF250	GTTCTTGTTAAAATTTGTCTATTTTAGC
USU101vacAdel_upF	GAAATTTTAGAGTGCATGCAAGATG
USU101vacAdel_upR	GTTAGTCACCCGGGTACCGAGCTCCATATCCACTTCAAGCTTGTTCC
USU101vacAdel_downF	CTAGAGTCGACCTGCAGGCATGCAAGTAATACCGCTCTTAAACCCATGC
USU101vacAdel_downR	GCATGTGGCTAGGTTGTTTTTCG
USU101vacA_FarupF	CGTTTAAAACAGCGTGTTTTAGG
USU101vacA_FardownR	GGTGTGCGTGAGCGTGGCGAGC
USU101vacALoc_comp_upR	CTCTCTGTCGAGAGTAGTGCGGCATGGGTTTAAGAGCGGTATTA
USU101vacALoc_comp_catF	TAATACCGCTCTTAAACCCATGCCGCACTACTCTCGACAGAGAG
USU101vacALoc_comp_catR	GCATGGGTTTAAGAGCGGTATTAGATAGATTTATGATATAGTGG
USU10vacALoc_comp_downF	CCACTATATCATAAATCTATCTAATACCGCTCTTAAACCCATGC
USU101_vacA_nt608_F	CCCCTTCTCAAAGTGGTGC
USU101_vacA_nt608_R	GCACCACTTTGAGAAGGGG
USU101_vacA_nt1366_F	GGCACTAGGTCAATCTTTTCTGG
USU101_vacA_nt1366_R	CCAGAAAAGATTGACCTAGTGCC
USU101_vacA_nt2119_F	GGTGGCAATACCACCAACTTACC
USU101_vacA_nt2119_R	GGTAAGTTGGTGGTATTGCCACC
USU101_vacA_nt2875_F	GGCGTGTATAGCCGTATCTTTGC
USU101_vacA_nt2875_R	GCAAAGATACGGCTATACACGCC
KanF	GAGCTCGGTACCCGGGTGACTAAC
KanR	CTTGCATGCCTGCAGGTCGACTCTAG
Cat_Mto5	CTGCTGTAAACTCAGTCCAAATACTCG
Cat_Mto3	CGAGTATTTGGACTGAGTTTACAGCAG
USU101vacAdel_KanF	GGAACAAGCTTGAAGTGGATATGGAGCTCGGTACCCGGGTGACTAAC
USU101vacAdel_KanR	GCATGGGTTTAAGAGCGGTATTACTTGCATGCCTGCAGGTCGACTCTAG

To construct the *cagA* restorant strain, three amplicons were generated: the sequence upstream including the *cagA* coding sequence using USU101 genomic DNA, the sequence approximately 1 kb downstream from the coding sequence using USU101 genomic DNA, and the *cat* cassette (to confer Cm resistance) using pCagCat pDNA (63). The amplicons from the three PCR reactions were joined together using SOE such that the upstream and *cagA* coding sequence amplicon flanked the downstream portion of the *cat* cassette and the downstream region of the *cagA* gene flanked the upstream portion of the *cat* cassette. The 1 kb sequence upstream of *cagA* along with the *cagA* CDS was amplified with USU101delcagA_upF and USU101_cagALoc_Comp_upR. The 1 kb sequence downstream of *cagA* was amplified with USU101_cagALoc_Comp_downF and USU101delcagA_downR. The ~750 bp *cat* cassette was amplified with USU101_cagALoc_Comp_catF and USU101_cagALoc_Comp_catR. The resulting 6 kb SOE product was gel-purified and further amplified to increase the amount of template for transformation. The resulting amplicon was used to naturally transform USU101. Transformants were selected on HBA plates supplemented with 6 µg/mL Cm. The Cm-marked *cagA* was confirmed using primers USU101cagA_FarupF and USU101cagA_FardownR, which reside outside of the cloned region. The correct insertion of the cat cassette was confirmed with primers cat_Mto5 and USU101cagA_FardownR, which revealed the cat cassette was in the reverse orientation and downstream of the *cagA* CDS. The resulting Cm-marked *cagA* USU101 strain was named DSM2078. Genomic DNA from strain DSM2078 was used to naturally transform strain DSM2076 (USU101 Δ*cagA*). Transformants were selected on HBA plates supplemented with 6 µg/mL Cm. Correct placement of the restorant *cagA* gene was verified with primers USU101cagA_FarupF and USU101cagA_FardownR, which lie outside the original cloned region. Additionally, the correct insertion of the cat cassette was confirmed with primers cat_Mto5 and USU101cagA_FardownR, which revealed the cat cassette was in the reverse orientation and downstream of the *cagA* CDS. The lack of the *aphA* gene for Kan^R^ was tested with primers KanF and KanR. The restored *cagA* gene sequence was verified with primers USU101cagAdel_upF, USU101cagAdel_downR, Cat_Mto3, Cat_Mto5, USU101_cagseq_nt960_F, USU101_cagseq_nt1400_R, USU101cagA1020R, USU101cagA1960F, and USU101cagAdel_upF250. The resulting restorant strain was named DSM2079.

To create the Δ*vacA* mutant strain, the sequence approximately 1 kb downstream of the *vacA* coding sequence and the sequence approximately 600 bp upstream plus 400 bp of the *vacA* coding sequence were amplified from USU101 genomic DNA along with the *aphA-3* gene from pUC18K-2 (62). Primer sequences used in this study are listed in Table 2. The 1 kb sequence upstream including a portion of *vacA* was amplified with USU101delvacA_upF and USU101delvacA_upR. The 1 kb sequence downstream of *vacA* was amplified with USU101delvacA_downF and USU101delvacA_downR. The kanamycin-resistant cassette encoded by the *aphA-*3 gene was amplified from pUC18K-2 with USU101vacAdel_KanF and USU101vacAdel_KanR. The three amplicons were joined using SOE such that the upstream and downstream fragments flanked the kanamycin-resistant cassette. The resulting amplicon was used to transform USU101 via natural transformation, and transformants were selected on HBA plates supplemented with 25 µg/mL kanamycin. The deletion of *vacA* was confirmed using primers USU101vacA_FarupF and USU101vacA_FardownR, which reside outside of the cloned region. The resulting strain was further confirmed by sequencing with primers USU101vacAdel_upF, USU101vacAdel_upR, USU101vacAdel_downF, and USU101vacAdel_downR, and the strain was named DSM2086.

To construct the *vacA* restorant strain, three amplicons were generated: the sequence 600 kb upstream plus the *vacA* coding sequence using USU101 genomic DNA, the sequence approximately 1 kb downstream from the coding sequence using USU101 genomic DNA, and the *cat* cassette using pCagCat pDNA (63) to confer Cm resistance. The amplicons from the 3 PCR reactions were joined together using SOE such that the upstream and *vacA* coding sequence amplicon flanked the downstream portion of the *cat* cassette and the downstream region of the *vacA* gene flanked the upstream portion of the *cat* cassette. The 600 bp sequence upstream of *vacA* along with the *vacA* CDS was amplified with USU101delvacA_upF and USU101_vacALoc_Comp_upR. The 1 kb sequence downstream of *vacA* was amplified with USU101_vacALoc_Comp_downF and USU101delvacA_downR. The ~750 bp *cat* cassette was amplified with USU101_vacALoc_Comp_catF and USU101_vacALoc_Comp_catR. The resulting 6 kb SOE product was gel-purified and further amplified. The resulting amplicon was used to naturally transform USU101. Transformants were selected on HBA plates supplemented with 6 µg/mL Cm. The Cm-marked *vacA* was confirmed using primers USU101vacA_FarupF and USU101vacA_FardownR, which reside outside of the cloned region. The correct insertion of the cat cassette was confirmed with primers cat_Mto5 and USU101vacA_FardownR, which revealed the *cat* cassette was in the reverse orientation and downstream of the *vacA* CDS. The resulting Cm-marked *vacA* USU101 strain was named DSM2087. Genomic DNA from strain DSM2087 was used to naturally transform strain DSM2086 (USU101 Δ*vacA*). Transformants were selected on HBA plates supplemented with 6 µg/mL Cm. Correct placement of the restorant *vacA* gene was verified with primers USU101vacA_FarupF and USU101vacA_FardownR, which lie outside the original cloned region. Additionally, the correct insertion of the *cat* cassette was confirmed with primers cat_Mto5 and USU101vacA_FardownR, which revealed the *cat* cassette was in the reverse orientation and downstream of the *vacA* CDS. The lack of the *aphA* gene for Kan^R^ was verified with primers KanF and KanR. The restored *vacA* gene sequence was verified with primers USU101delvacA_upF, USU101vacAdel_upR, USU101delvacA_downF, USU101delvacA_downR, Cat_Mto3, Cat_Mto5, USU101vacA_FarupF, USU101vacALoc_comp_upR, USU101vacA_FardownR, USU101_vacA_nt608_F, USU101_vacA_nt608_R, USU101_vacA_nt1366_F, USU101_vacA_nt1366_R, USU101_vacA_nt2119_F, USU101_vacA_nt2119_R, USU101_vacA_nt2875_F, and USU101_vacA_nt2875_R. The resulting restorant strain was named DSM2088.

### CagA Western blot analysis

*H. pylori* strains were cultured on blood agar plates and resuspended in water. The bacterial suspensions were standardized by OD_600_, boiled in Laemmli sample buffer, and then loaded onto a 10% SDS-PAGE gel. After electrophoresis and transfer of proteins to nitrocellulose paper, the membrane was blocked overnight with 2% milk TBST (Tris-buffered saline [TBS] with 1% Tween 20) and probed with a 1:5,000 dilution of anti-CagA rabbit serum (Santa Cruz Biotechnology, Dallas, TX, USA). The blot was then probed with a 1:5,000 dilution of anti-rabbit IgG horseradish peroxidase conjugate (Promega, Madison, WI, USA) and developed using SuperSignal West Pico plus chemiluminescent substrate (Thermo Scientific).

### VacA Western blot analysis

*H. pylori* strains were cultured in Brucella broth containing supplemental cholesterol. Culture supernatants were collected, boiled with Laemmli sample buffer, and then loaded onto a 10% SDS-PAGE gel. After electrophoresis and transfer of proteins to nitrocellulose paper, the membrane was blocked overnight with 2% milk TBST and probed with anti-VacA rabbit antiserum 958 (64). The blot was then probed with anti-rabbit IgG horseradish peroxidase conjugate (Promega) and developed using SuperSignal West Pico plus chemiluminescent substrate (Thermo Scientific).

### Vacuolation assay

HeLa cells were cultured in minimal essential medium containing 10% fetal bovine serum. The cells were seeded in 96-well flat-bottom tissue culture-treated plates at 1 × 10^5^ cells/well and grown for 24 hours. Bacterial culture supernatants were serial diluted and added to cells in medium supplemented with 5 mM ammonium chloride. The next day, neutral red dye was added to cells for 30 minutes. Cells were then washed three times with 0.9% saline. Acid alcohol (97% ethanol, 3% HCl) was added to extract dye, and then optical density at 540 nm was measured (65).

### CagA translocation

The translocation of CagA was assessed using previous methodologies (66). In brief, AGS cells were seeded (5 × 10^5^ cells per well) in 6-well plates. On the following day, the cells were co-cultured with *H. pylori* for 6 hours with a multiplicity of infection (MOI) of 100:1. The translocation of CagA was detected using an anti-phosphotyrosine (anti-PY99) mouse monoclonal antibody (Santa Cruz Biotechnology). After stripping the membranes, CagA was detected by using an anti-CagA rabbit polyclonal antibody (Santa Cruz Biotechnology).

### IL-8 induction

AGS cells were cultured in RPMI medium containing 10% fetal bovine serum and seeded at 2.5 × 10^4^ cells per well in a 96-well plate. The following day, *H. pylori* was added at a MOI of 100:1, and co-cultures were incubated for 4 hours at 37°C with 5% CO_2_. The co-culture supernatants were collected and IL-8 was quantified using a human CXCL8 enzyme-linked immunosorbent assay (R&D Systems, Minneapolis, MN, USA) (67).

### Gerbil infections and histology

Five- to eight-week-old male (infections conducted at Uniformed Services University) or female (infections conducted at University of South Carolina School of Medicine) Mongolian gerbils (Charles River Laboratories, Inc., Wilmington, MA, USA) were infected with 10^9^ *H. pylori* bacteria as previously described (30, 68). Briefly, gerbils were fasted for 2 hours prior to infection with 500 µL of liquid-grown *H. pylori* cells. For the initial gerbil experiments, five animals each were infected via orogastric gavage with one of the following strains: DU3, G1, GU12, DSM719, USU101, or J166; the animals were sacrificed, and the stomachs were harvested at 1 or 3 months post-infection. In the next gerbil experiment, five animals each were infected via orogastric gavage with either USU101 or J166 bacterial cells for each timepoint. The gerbils were sacrificed at 1, 2, 3, 4.5, or 6 months following infection, and the stomachs were harvested. For the experiment conducted at another institution, USU101 was administered via oral gavage to 15 gerbils, and stomachs were harvested from five gerbils each at 4, 8, and 16 weeks following infection. For subsequent gerbil infections, four to five animals each were orogastrically infected with either the WT USU101, Δ*cagA*, Δ*vacA*, *cagA* restorant, or *vacA* restorant strains for each timepoint. Additionally, a biologically independent repeat of this experiment was conducted with similar numbers of animals. At 1, 2, or 3 months post-infection, gerbils were sacrificed, and the glandular portion of the stomach was harvested for colony enumeration and pathological examination. For all animal experiments, the harvested stomachs were bisected, and one portion was homogenized with a Bullet Blender (Next Advance, Troy, NY, USA) using 1.6 mm stainless steel beads, and the resulting homogenate was plated on HBA plates supplemented with 10 µg/mL nalidixic acid and 100 µg/mL bacitracin for CFU determination. The remaining stomach portion was placed in 10% neutral buffered formalin (Epredia, Inc., Kalamazoo, MI, USA) and subsequently embedded in paraffin, sectioned, and stained with hematoxylin and eosin. The stained sections were blinded and scored for inflammation by a pathologist and graded based on gastric pathology for disease and cancer development. Acute and chronic inflammation were each graded on a 0–3 scale (0, none; 1, mild; and 2, moderate; 3, marked) in the gastric antrum and corpus separately. Acute inflammation was defined as infiltration of neutrophils and chronic inflammation as infiltration of mononuclear leukocytes. Acute inflammation and chronic inflammation in antrum and corpus were combined for a maximum inflammation score of 12 for each animal. As indicated above, two independent biological replicates of the final gerbil infection experiment were performed.
